# Human-caused habitat fragmentation can drive rapid divergence of male genitalia

**DOI:** 10.1111/eva.12223

**Published:** 2014-10-31

**Authors:** Justa L Heinen-Kay, Holly G Noel, Craig A Layman, R Brian Langerhans

**Affiliations:** 1 Department of Biological Sciences and W. M. Keck Center for Behavioral Biology, North Carolina State UniversityRaleigh, NC, USA; 2 Department of Applied Ecology, North Carolina State UniversityRaleigh, NC, USA

**Keywords:** anthropogenic environmental change, genital evolution, gonopodium, human-induced phenotypic change, natural selection, Poeciliidae, sexual selection

## Abstract

The aim of this study rests on three premises: (i) humans are altering ecosystems worldwide, (ii) environmental variation often influences the strength and nature of sexual selection, and (iii) sexual selection is largely responsible for rapid and divergent evolution of male genitalia. While each of these assertions has strong empirical support, no study has yet investigated their logical conclusion that human impacts on the environment might commonly drive rapid diversification of male genital morphology. We tested whether anthropogenic habitat fragmentation has resulted in rapid changes in the size, allometry, shape, and meristics of male genitalia in three native species of livebearing fishes (genus: *Gambusia*) inhabiting tidal creeks across six Bahamian islands. We found that genital shape and allometry consistently and repeatedly diverged in fragmented systems across all species and islands. Using a model selection framework, we identified three ecological consequences of fragmentation that apparently underlie observed morphological patterns: decreased predatory fish density, increased conspecific density, and reduced salinity. Our results demonstrate that human modifications to the environment can drive rapid and predictable divergence in male genitalia. Given the ubiquity of anthropogenic impacts on the environment, future research should evaluate the generality of our findings and potential consequences for reproductive isolation.

## Introduction

Humans are altering ecosystems worldwide, but the evolutionary consequences of such impacts are poorly understood (Palumbi 2001a; Smith and Bernatchez 2008; Sih et al. 2011). A wide range of human activities can lead to altered selection regimes experienced by native organisms, including introduction or removal of predators, habitat loss or alteration, and changes in nutrient availability and abiotic conditions. These novel selection regimes can elicit rapid changes in the phenotypes of organisms living in human-altered environments (Palumbi 2001b; Stockwell et al. 2003; Hendry et al. 2008). One particularly widespread human-induced environmental impact, anthropogenic habitat fragmentation, has garnered much attention for contributing to species extinctions and loss of biodiversity (Saunders et al. 1991; Fahrig 2003; Foley et al. 2005; Fischer and Lindenmayer 2007), but its effects on phenotypic diversification and speciation are understudied (Hendry et al. 2011). Most evolutionary work on habitat fragmentation has focused on effects of reduced population sizes, reduced genetic diversity, and reduced gene flow among populations (Fahrig 2003; Ewers and Didham 2006; Blanchet et al. 2010). Yet fragmented environments often exhibit dramatically different ecological conditions than unfragmented environments, suggesting that changes in selection might often drive phenotypic shifts. To better understand how ecological consequences of habitat fragmentation might impact natural populations, we need to investigate how predictably fragmentation might lead to phenotypic change (Franssen et al. 2013).

Changes in environmental conditions (e.g., predators, parasites, abiotic factors) commonly alter the strength or mode of sexual selection in diverse taxa (Seehausen et al. 1997; Zuk and Kolluru 1998; Scordato et al. 2012; Arbuthnott et al. 2014). Sexually selected traits can evolve very rapidly (Zuk et al. 2006) often at faster rates than nonsexually selected characters (Arnegard et al. 2010; Gonzalez-Voyer and Kolm 2011). Thus, sexual traits might commonly diversify in response to human-induced environmental changes (Seehausen et al. 1997; Candolin et al. 2007). One sexually selected trait that may prove especially susceptible to human impacts is male genital morphology. Biologists have long recognized the remarkable diversity of male genital morphology, which is often regarded as the most rapidly evolving trait in internally fertilizing animals (Dufour 1844; Eberhard 1985). Researchers agree that postcopulatory sexual selection (cryptic female choice, sperm competition, sexual conflict) bears much responsibility for this phenomenon (Arnqvist 1998; Hosken and Stockley 2004; Rowe and Arnqvist 2011), but other mechanisms may prove important as well (Langerhans 2011; Simmons 2014).

We suggest that rapid human-induced environmental change might promote genital diversification, but to our knowledge, this has never before been explored. Our hypothesis reflects the logical conclusion of three well-supported premises: (i) human impacts alter the environment, (ii) ecological variation frequently influences the nature or strength of sexual selection, and (iii) sexual selection drives rapid and divergent evolution of male genital morphology. Understanding whether human impacts may ultimately drive genital diversification is critical because it carries important consequences for fitness, gene flow among populations, and the formation of new species. For instance, variation in male genital shape and size affects insemination and fertilization success in various taxa (Arnqvist and Danielsson 1999; Evans et al. 2011; Simmons 2014). Moreover, genital incompatibilities between populations can provide mechanical or sensory obstacles to successful reproduction in internally fertilizing animals, with differences in genital shape being implicated in speciation (Kamimura and Mitsumoto 2012; Kubota et al. 2013; Wojcieszek and Simmons 2013).

Multiple aspects of male genital morphology—for example, size, allometry, shape—exhibit intriguing evolutionary patterns, but virtually nothing is known about how human-induced environmental changes may affect genitalia. Male genital size and shape can evolve rapidly in response to variation in ecological conditions via altered natural or sexual selection (Langerhans et al. 2005; Evans et al. 2011; Langerhans 2011; Heinen-Kay and Langerhans 2013). For instance, natural selection may act directly on nonretractable genitalia via effects on locomotor ability (Langerhans et al. 2005). Predation risk is well known for its influence on sexual selection—greater predation risk tends to favor shorter copulation duration with reduced courtship and increased sexual conflict (Magnhagen 1991; Magurran and Seghers 1994; Rowe et al. 1994; Heinen et al. 2013). This may in turn select for particular genital morphologies that enhance sperm transfer rate or fertilization success under these conditions. In addition to genital size and shape, allometry of male genitalia has been well studied. In contrast with many sexually selected traits (Kodric-Brown et al. 2006), male genitalia typically exhibit a negative static allometric (hypoallometric) relationship with body size (Eberhard et al. 1998, 2009; Eberhard 2009) where small males often have disproportionally large genitalia while large males often have disproportionally small genitalia. Whether environmental variation might lead to changes in genital scaling is unknown, but shifts in directional or stabilizing selection on genital size across environments can alter genital scaling (Eberhard et al. 2009).

Here, we investigate whether the ecological consequences of recent habitat fragmentation have led to divergence in multiple aspects of male genital morphology within three livebearing fish species endemic to the Bahamas. Fragmentation of tidal creeks is pervasive across the Bahama Archipelago and can alter the selective regimes of resilient species that persist in the altered conditions (e.g., Valentine-Rose et al. 2007a,b, 2011; Valentine-Rose and Layman 2011; Araujo et al. 2014). By examining three species across six different islands, we test whether human-induced tidal-creek fragmentation has resulted in (i) no phenotypic change, (ii) parallel changes in male genital morphology across species and islands, (iii) nonparallel changes, or (iv) some combination of these patterns. We additionally attempt to pinpoint particular environmental agents most strongly associated with observed morphological divergence between fragmentation regimes.

### Study system and predictions

Male livebearing fishes in the genus *Gambusia* (mosquitofishes, family Poeciliidae) transfer sperm internally to females both cooperatively and coercively using a nonretractable, modified anal fin called the gonopodium. Gonopodium size varies greatly across poeciliid species, ranging from 20% to 70% of the body length (Rosen and Gordon 1953; Langerhans 2011), and is subject to trade-offs between natural and sexual selection. Females of multiple *Gambusia* species prefer males possessing larger gonopodia (Langerhans et al. 2005; Kahn et al. 2010). However, larger gonopodia create more frictional drag, which hinders swimming performance while escaping predatory attacks; indeed, males tend to exhibit smaller gonopodia in high-risk populations, and males with smaller gonopodia experience higher survivorship in the presence of predators (Langerhans et al. 2005; Langerhans 2011). Little work has examined gonopodial allometry in *Gambusia* fishes, although female preferences for large gonopodia in some species, and prior work in other poeciliid fishes (Kelly et al. 2000; Jennions and Kelly 2002), suggest that positive allometry, where gonopodium size increases disproportionately with body size, is possible.

The distal tip of the gonopodium is highly differentiated (comprising hooks, spines, serrae, etc.) and directly contacts the female during copulation (Rosen and Gordon 1953). Unlike gonopodium size, the distal tip of the gonopodium appears unimportant for both premating sexual selection and locomotor performance-based natural selection owing to its very small size (<1 mm). However, gonopodial distal-tip shape has been demonstrated to affect sperm transfer during forced copulations in another livebearing fish species (Evans et al. 2011; Kwan et al. 2013) and might prove important during postcopulatory sexual selection such as sperm competition and cryptic female choice (Evans and Pilastro 2011; Gasparini et al. 2011; Langerhans 2011). Notably, prior work found divergence of gonopodial distal-tip shape between predatory environments in *Gambusia* (Heinen-Kay and Langerhans 2013), confirming that ecological variation is capable of driving evolution of distal-tip shape.

**Figure 1:**
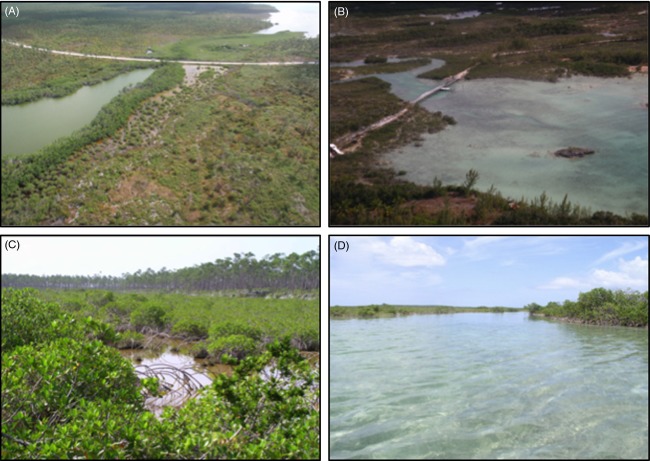
Representative aerial photographs of (A) fragmented, (B) unfragmented, and ground-level photographs of (C) fragmented and (D) unfragmented Bahamian tidal creeks. The road crossing in (A) severely restricts hydrological connectivity, while the bridge in (B) provides minimal to no restriction of water flow.

Three species of *Gambusia* are endemic to the Bahama Archipelago and commonly inhabit ‘tidal creeks’: (i) *Gambusia hubbsi* Breder 1934 is known to inhabit the northwestern islands of the Great Bahama Bank (Bimini, Berry Islands, Andros, New Providence), (ii) *Gambusia manni* Hubbs 1927 appears to inhabit all other islands within the Great Bahama Bank, as well as overlap with *G. hubbsi* on New Providence (but not sympatric within tidal creeks), and (iii) a yet unnamed species, *Gambusia* sp., inhabits the islands of the Little Bahama Bank (see Supporting Information: *Focal species*). Bahamian tidal creeks are shallow, tidally influenced systems typically characterized by a relatively narrow creek mouth that broadens landward to wetlands dominated by *Rhyzophora mangle* (red mangrove). Most of the water flux in these systems arises from tidal exchange (freshwater input only provided via rainfall and aquifer percolation), so salinities in unfragmented systems are typically around 35 ppt and the biotic communities comprise marine taxa (Layman et al. 2004; Valentine-Rose et al. 2007a,b). Tidal-creek fragmentation represents one of the most widespread human-caused environmental impacts in nearshore waters of the Bahamas (e.g., over 80% of tidal creeks are fragmented on Andros Island; Layman et al. 2004) and is generally caused by roads constructed across tidal creeks without proper flow conveyance structures such as culverts or bridges (Fig. 1). This greatly reduces hydrological connectivity, that is, the water-mediated transfer of matter, energy, or organisms within or between elements of the hydrological cycle (Pringle 2001, 2003a,b). Most of the roads that fragment tidal creeks throughout the Bahamas were constructed between the late 1950s and early 1970s. Once isolated from the ocean, fragmented tidal creeks have considerably reduced tidal amplitude (typically 0–5 cm tidal amplitude) compared with unfragmented (natural) tidal creeks (typically 50–80 cm tidal amplitude), leading to greater extremes in abiotic factors, reduced animal biomass, and changes in community composition (Valentine-Rose et al. 2007a,b, 2011; Valentine-Rose and Layman 2011). Mosquitofish inhabit both fragmented and unfragmented tidal creeks, but occur in far greater densities in fragmented sites (Layman et al. 2004; Valentine-Rose et al. 2007b; Araujo et al. 2014). Unfragmented tidal creeks contain much higher densities of piscivorous fishes, including predators of *Gambusia* such as *Sphyraena barracuda* Edwards 1771 (great barracuda), *Strongylura* spp. (needlefish), and *Lutjanus* spp. (snappers; Layman et al. 2004; Araujo et al. 2014).

Because fragmented tidal creeks exhibit a drastic reduction in predatory fish densities and a considerable increase in *Gambusia* densities, we predict that patterns of gonopodial divergence should mirror patterns of genital evolution in *G. hubbsi* (a focal species in the current study) inhabiting Bahamian blue holes where similar ecological differences over thousands of years have resulted in divergent evolution of male genital morphology. Specifically, we predict smaller gonopodia with more elongate and bony distal tips in unfragmented tidal creeks and larger gonopodia with more rounded distal tips possessing greater areas of soft tissue in fragmented tidal creeks (Langerhans et al. 2005; Heinen-Kay and Langerhans 2013). Differences in gonopodium size are believed to derive from a trade-off between attracting females and avoiding predation. Gonopodial distal-tip shape may diverge because of stronger sexual conflict or postmating sexual selection in high-predation sites, where elongate and rigid tips enhance insemination efficiency or increase fertilization probability during the rapid and frequent copulation attempts in this risky environment (Godin 1995; Heinen et al. 2013). Gonopodial meristics (number of serrae and spines) may also influence a male's ability to ‘anchor’ to or stimulate the female during copulation and thus might also differ between fragmentation regimes in response to differential sexual selection. Premating sexual selection appears stronger in low-predation environments where forceful sexual behaviors are less frequent (Heinen et al. 2013). In this low-risk environment, females may exert greater premating choice, potentially reducing the strength of postmating selection on genital morphology. Whether gonopodia exhibit negative allometric scaling typical of genitalia in other previously studied organisms (mostly insects and spiders; Eberhard 2009) is an empirical question. We predict that the allometric scaling of male genitalia will not differ between fragmentation regimes because allometric relationships tend to remain fairly constant over short evolutionary time frames (Voje et al. 2014), and we do not hypothesize differences in selection between fragmentation regimes that should alter genital allometry (Eberhard et al. 2009). Although our primary predictions for genital divergence between fragmented and unfragmented tidal creeks rest on differences in predatory fish density, a suite of other environmental factors, including salinity, turbidity, pH, and dissolved oxygen, may also differ between fragmentation regimes and could contribute to genital divergence. Each of these abiotic factors has previously been demonstrated to impact sexual selection in other fish systems (salinity: McCairns et al. 2011; turbidity: Heuschele et al. 2009; Sundin et al. 2010; dissolved oxygen: Jones and Reynolds 1999; pH: Sundin et al. 2013). Overall, we expect that each species will exhibit species-specific genital features, but that parallel responses to fragmentation across all three species should occur if generalized consequences of habitat fragmentation across the Bahamas repeatedly generate similar selection pressures.

## Materials and methods

### Field sampling

**Figure 2:**
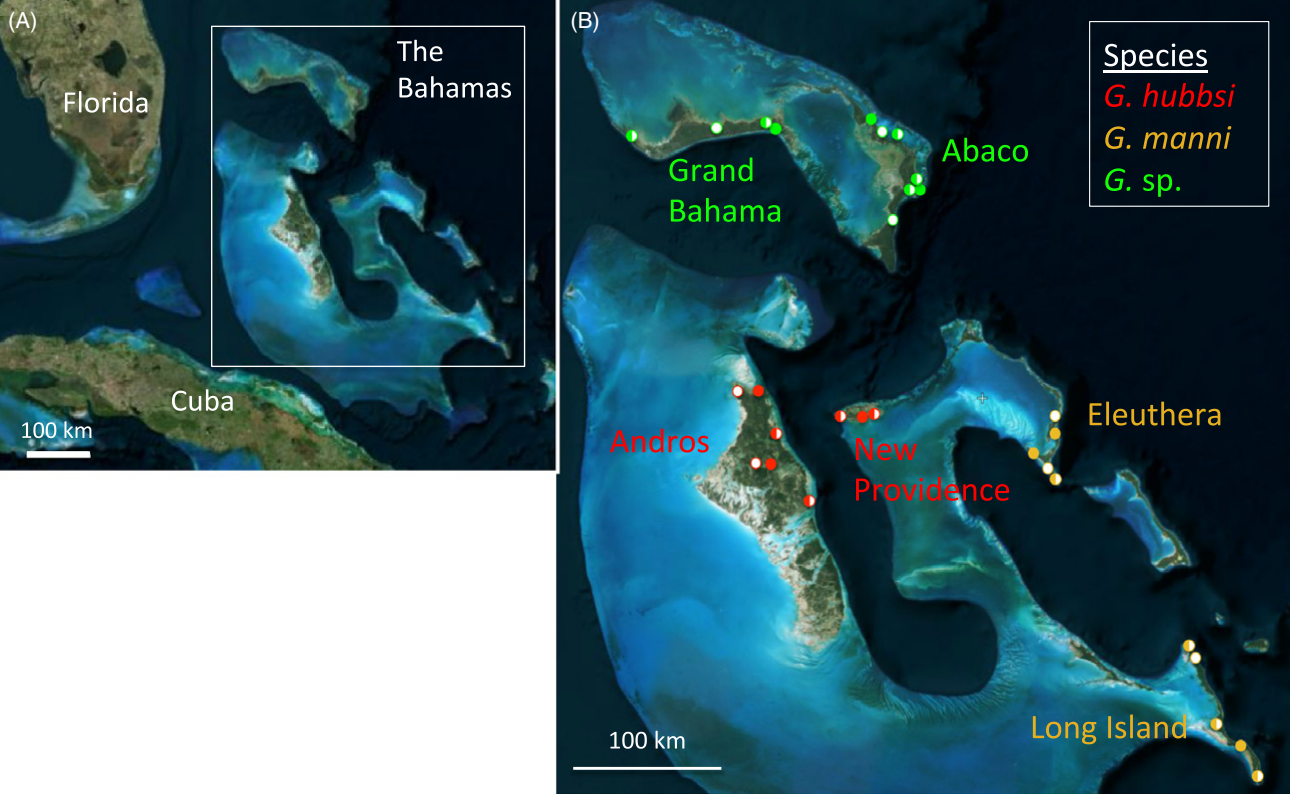
Map of (A) the study region in the northwestern Bahamas and (B) specific tidal creek collection localities for each *Gambusia* species on each island. Open circles: fragmented tidal creeks, filled circles: unfragmented tidal creeks, half-filled circles: both fragmented and unfragmented sites in close proximity.

We collected *Gambusia* from 43 tidal creeks (21 fragmented; 22 unfragmented) across six Bahamian islands (Abaco, Andros, Eleuthera, Grand Bahama, Long Island, and New Providence) in March–April 2010 using dip nets and minnow traps. For each species, we collected multiple populations from both fragmented and unfragmented tidal creeks on two separate islands (Fig. 2; Table S2) and immediately preserved fish on site in 95% ethanol.

Simultaneous with fish collections, we measured a suite of six environmental factors selected based on their potential influence on genital diversification: density of piscivorous fish, mosquitofish density, salinity, pH, turbidity, and dissolved oxygen. We conducted all measurements at each locality with the exception that *Gambusia* density was not measured at one site (South Beach Creek, New Providence) due to concern that reduced visibility might bias the survey. We measured density of piscivorous fish using underwater visual census (UVC) methods (Nagelkerken et al. 2000; Layman et al. 2004), which provide reliable estimates of relative predator densities in Bahamian tidal creeks (Layman et al. 2004; Valentine-Rose et al. 2007a, 2011). In sites with water too shallow to employ UVC, we conducted predator surveys from above the water by slowly walking the survey areas (all piscivores are readily visible from above in these clear waters). We conducted surveys covering approximately 3000 m^2^ within each creek, encompassing the region where we collected *Gambusia*. We included the following piscivorous fish species in our counts: barracuda, needlefish, snappers, tarpon (*Megalops atlanticus* Valenciennes 1847), Nassau grouper (*Epinephelus striatus* Bloch 1792)*,* jacks (*Caranx* spp.), and lionfish (*Pterois* spp.). Nearly all of the predators encountered in our surveys comprised known predators of *Gambusia*—barracuda, needlefish, and snappers (>99%)—with other potential predators rarely observed. We used our survey counts to calculate piscivore density (#/km^2^) for each site. We measured mosquitofish density using quadrat surveys, in which we counted all *Gambusia* individuals within 20 0.25 m^2^ quadrats within each tidal creek. We randomly chose quadrat locations across the microhabitats where *Gambusia* were observed, with all quadrats >2 m apart. For these surveys, the observer approached each predesignated area, stood still 1 m from the quadrat location, and waited 1 min prior to counting fish. We used the average of these 20 quadrat counts to calculate our estimate of *Gambusia* density (#/m^2^) for each site. This method proved highly effective due to water clarity and these fishes' general tendency to be undisturbed by our presence. We measured salinity and dissolved oxygen using a YSI 85 handheld multiparameter meter (Yellow Springs, OH, USA), water turbidity using an Oakton T-100 turbidimeter (Vernon Hills, IL, USA), and pH using a Hanna HI 98128 handheld meter (Carrollton, TX, USA). All six ecological parameters were measured multiple times over multiple years within a subset of tidal creeks to estimate repeatability—all parameters exhibited significant repeatability, confirming that our snapshot estimates provided meaningful estimates for comparison among sites (Table S3).

### Gonopodium size and allometry

We captured whole-body lateral photographs of each fish (*n* = 410; Table S2) using a Canon Rebel XS digital camera (Canon Inc., Melville, NY, USA). Using tpsDig2 software (Rohlf 2010a), we measured standard length (tip of rostrum to posterior tip of hypural plate) and gonopodium lateral surface area (area inside the gonopodium's outer boundaries, including anal fin rays 1–5) from these photographs (Fig. S2). Although some previous work on gonopodial variation in poeciliids focused on gonopodium length (Kelly et al. 2000; Jennions and Kelly 2002; Kahn et al. 2010), we measured surface area because our hypothesis of divergent selection between fragmentation regimes centers on effects of gonopodium surface area, rather than length, on fitness components (i.e., mating preferences for lateral surface area, frictional drag incurred by gonopodia during locomotion; Langerhans et al. 2005). Both measurements were log_10_-transformed to meet assumptions of normality of residuals in statistical analyses.

Because we wished to examine the consistency of phenotypic differences between fragmentation regimes across species and islands, we employed a statistical approach that can explicitly test for and quantify both shared and unique effects of fragmentation on male genital morphology across these groups (see Langerhans and DeWitt 2004). We employed this approach with each aspect of male genital morphology (see below). All analyses were performed in JMP (SAS Institute, Cary, NC, USA) unless otherwise noted. Prior to tests of male genital morphology, we first conducted a test to confirm that body size and fragmentation status were not confounded. We performed a general linear mixed model (GLMM) where log_10_-transformed standard length served as the response variable, fixed effects included fragmentation status, species, island nested within species, and all two-way interactions with fragmentation status, while population served as a random effect.

To test for shared and unique responses of gonopodium size to tidal-creek fragmentation across the species and islands, we conducted a GLMM as described above, but with log_10_-transformed gonopodium area as the response variable, and additional model terms for log_10_-transformed standard length (covariate to control for allometry) and the interaction between log_10_-transformed standard length and fragmentation status (test for allometric differences between fragmentation regimes). Because we observed significant heterogeneity of slopes across fragmentation regimes (i.e., significant interaction between standard length and fragmentation), we could not simply interpret differences in relative gonopodium size—rather, the key source of variation involved allometry. Thus, we conducted analyses specifically designed to test for among-population variation in allometric slopes.

To directly investigate potential differences in scaling relationships among populations, we calculated allometric slopes for each population with a sample size >5 (due to reduced confidence in slopes calculated with <5 data points). This excluded three populations. Because it remains unclear whether slopes calculated using ordinary least squares (OLS) or reduced major axis (RMA) regression is most appropriate for allometric studies (Voje et al. 2014), we employed both methods. We then weighted our estimates of allometric slopes by population sample size in an effort to give more weight to more precise estimates. We conducted two general linear models (GLM) to test for shared and unique effects of habitat fragmentation on gonopodial allometry, with population slopes (OLS and RMA slopes, separately) serving as the response variable, and our five major terms of interest serving as independent variables: fragmentation status, species, island nested within species, fragmentation status × species, and fragmentation status × island nested within species.

### Gonopodial distal-tip shape

To examine variation in gonopodial distal-tip shape, we photographed the lateral, left side of each gonopodial distal tip using a Leica S8 APO stereoscope equipped with a DFC 425 digital camera and a TL RCI base (Leica Microsystems Inc., Buffalo Grove, IL, USA). We captured three to five images of each gonopodial distal tip at 128× magnification and stacked the photographs to comprise one composite image using Helicon Focus software (http://www.heliconsoft.com/). Gonopodial distal-tip shape was quantified for 235 fish (Table S2) using geometric morphometric methods (Zelditch et al. 2004; Heinen-Kay and Langerhans 2013). Sample sizes for gonopodial surface area and distal-tip shape differ because preservation effects and natural variation precluded photographs adequate to examine both features on all specimens. Using tpsDig2 software, we digitized 44 homologous landmarks (Fig. S3) chosen to provide adequate representation across the gonopodial distal tip. Textual descriptions of landmark locations can be found in Heinen-Kay and Langerhans (2013); landmarks 38–40 and 50–51 from Heinen-Kay and Langerhans (2013) were not included in the present study because they exhibited unusually high variance owing to differences among species (i.e., ∼2.5 × greater standard deviation than other landmarks). Using tpsRelw software (Rohlf 2010b), we extracted relative warps (principal components of shape variation) to reduce dimensionality of the data. We retained the first 15 relative warps for use in analyses, which explained 89.6% of shape variance. Centroid size (square root of the summed, squared distances of all landmarks from their centroid) provided an estimate of gonopodial distal-tip size for analyses.

We conducted a mixed-model multivariate analysis of covariance (mancova) to test for shared and unique effects of habitat fragmentation on gonopodial distal-tip shape across species and islands. The 15 relative warps served as response variables. Log-transformed gonopodial distal-tip centroid size, residual gonopodium size (from log-log regression of surface area on standard length), and log-transformed standard length were included as covariates to control for multivariate allometry, as all three factors could exhibit allometric effects on gonopodial distal-tip shape. These three potential sources of allometry exhibited low multicollinearity (all variance inflation factors <3.9); VIFs <10 are not considered problematic (Myers 1990). Other predictors included the five terms of interest described above for the allometric slope models, and population served as a random effect. Data met assumptions of mancova. An *F* test based on Wilks's Λ was used to determine statistical significance for the covariates, whereas we conducted an *F* test employing restricted maximum likelihood and the Kenward–Roger degrees of freedom adjustment (Kenward and Roger 1997) using the MIXED procedure in SAS to test significance of all other terms. This latter procedure enabled us to employ population as the unit of replication, effectively treating it as a random effect (Hassell et al. 2012; Heinen-Kay and Langerhans 2013; Riesch et al. 2013; Martin et al. 2014). To determine the relative importance of each predictor in our model, we calculated Wilks's partial *η*^2^ as an estimate of multivariate effect size (Langerhans and DeWitt 2004).

To obtain a single value that described gonopodial distal-tip shape for each individual, we calculated scores along **d**, the divergence vector, by conducting a principle component analysis on the sums of squares cross product matrix for the fragmentation status term in the mancova (Langerhans 2009). **d** is a multivariate axis summarizing the greatest variation in gonopodial distal-tip shape between fragmentation regimes while controlling for other model terms (Langerhans 2009). Thus, instead of examining variation in each relative warp individually, **d** provides a single axis that summarizes the major differences in gonopodial distal-tip shape between fragmentation regimes (i.e., linear combination of relative warps that exhibit greatest correlation with fragmentation regime). We used this multivariate axis to visualize gonopodial distal-tip shape differences between fragmentation regimes in tpsRelw. Individual scores along **d** were employed in remaining analyses involving gonopodial distal-tip shape.

### Gonopodial distal-tip meristics

In addition to investigating potential divergence in multivariate gonopodial distal-tip shape, we wished to discover whether any traditional meristic characters on the distal tip have shifted as a result of habitat fragmentation. We counted the number of serrae on gonopodial fin ray 4a and the number of spines on gonopodial fin ray 3 (Fig. S3) from distal-tip photographs (*n* = 235). We employed GLMs for analysis of meristics because the counts (and model residuals) met assumptions of normality. We investigated shared and unique effects of habitat fragmentation on gonopodial distal-tip meristics across species and islands using separate GLMMs. Model terms included in each model mirrored that described above for gonopodial distal-tip shape (i.e., three covariates to control for multivariate allometry, five major terms of interest, and population as a random effect).

### Model selection of ecological factors

We employed model selection to pinpoint particular environmental agents most strongly associated with observed divergence in gonopodial features between fragmentation regimes. Prior to model selection, we first conducted GLMs with each of the following environmental factors serving as response variables to determine the consistency of ecological differences between fragmentation regimes: piscivore density, *Gambusia* density, salinity, turbidity, pH, and dissolved oxygen. For each environmental factor, our model included our five terms of interest. Consistent differences between fragmentation regimes were evident for piscivore density, *Gambusia* density, and salinity and were suggestive for turbidity (Table S4). Thus, these four environmental factors served as possible predictors in our model selection analyses. For each gonopodial trait exhibiting significant differences between fragmentation regimes, we conducted model selection using GLMs with population mean values for the gonopodial feature of interest as response variables, the four noted environmental variables as potential independent variables, and forced inclusion of terms for species and island nested within species. The latter terms were included to control for variation among species and islands, thus allowing us to focus specifically on variation within islands. Multicollinearity was low in all models (all VIFs <2.7), allowing us to differentiate between competing hypotheses. For each trait, we considered models with Δ AIC_*c*_ scores <2 as selected models (Burnham and Anderson 2002).

## Results

### Gonopodium size and allometry

Body size only exhibited significant differences between islands (*F*_3,32.29_ = 3.24, *P* = 0.0348), confirming that fragmentation status was not confounded with body size (*F*_1,32.5_ = 0.85, *P* = 0.3626; all other terms, *P* > 0.24). Male *Gambusia* from fragmented tidal creeks exhibited a marginally nonsignificant trend toward possessing smaller gonopodia than counterparts in unfragmented sites (Table 1). However, significant heterogeneity of slopes indicated differences in allometry between fragmentation regimes. Thus, effects of fragmentation on gonopodium size appeared to largely influence allometry (slope), not simply relative gonopodium size (intercept).

**Table 1:** Results of general linear mixed model examining variation in log-transformed gonopodium surface area in three closely related *Gambusia* species inhabiting six Bahamian islands

Source	*F*	df	*P*
Standard length (SL)	1559.33	1, 391.7	<0.0001
Fragmentation status (Frag)	3.74	1, 33.09	0.0618
Species	0.96	2, 33.09	0.3946
Frag × Species	0.71	2, 33.09	0.4992
Island [Species]	2.99	3, 33.34	0.0449
Frag × Island [Species]	0.18	3, 33.34	0.9123
Frag × SL	6.29	1, 391.7	0.0125

**Figure 3:**
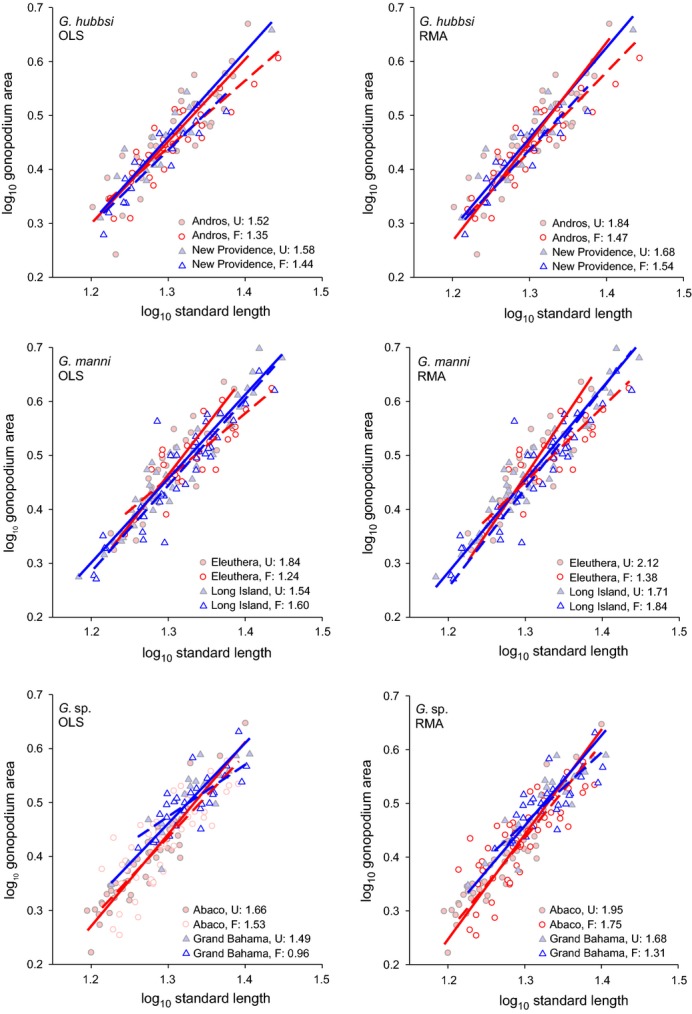
Variation in gonopodial allometry between fragmented and unfragmented tidal creeks on each island. Open symbols and dashed lines: fragmented tidal creeks (F); filled symbols and solid lines: unfragmented tidal creeks (U). Ordinary least squares regression (OLS) on the left and reduced major axis regression (RMA) slopes on the right.

Explicit examination of mean population allometric slopes revealed significant differences in allometry between fragmented and unfragmented tidal creeks using both ordinary least squares and reduced major axis regression approaches (Table 2). Populations in fragmented tidal creeks tended to exhibit shallower allometric slopes than those in unfragmented sites (Fig. 3). Gonopodial allometry also varied among islands (Table 2; Fig. 3). Model selection pointed to *Gambusia* density and salinity as important explanatory factors describing variation in gonopodial allometric slopes (Table 3). The top model included only *Gambusia* density (OLS: *F*_1,33_ = 5.06, *P* = 0.0313, *η*^2^ = 9.58%; RMA: *F*_1,33_ = 4.02, *P* = 0.0532, *η*^2^ = 8.20%), while the second best model included only salinity (OLS: *F*_1,33_ = 4.39, *P* = 0.0439, *η*^2^ = 8.97%; RMA: *F*_1,33_ = 2.98, *P* = 0.0935, *η*^2^ = 6.29%). These environmental factors tended to explain 50% (salinity) to 60% (*Gambusia* density) as much variance as a term for ‘fragmentation status,’ suggesting that much of the observed allometric differences between fragmentation regimes ultimately derived from some aspects of *Gambusia* density and salinity.

**Table 2:** Results of separate general linear models examining variation in gonopodial allometric slopes weighted by sample size and calculated using both ordinary least squares (OLS) and reduced major axis (RMA) regression

		OLS		RMA	
Source	df	*F*	*P*	*F*	*P*
Fragmentation status (Frag)	1, 28	7.12	0.0125	6.03	0.0205
Species	2, 28	1.53	0.2349	1.50	0.2398
Frag × Species	2, 28	0.65	0.5297	0.32	0.7318
Island [Species]	3, 28	4.37	0.0121	3.78	0.0214
Frag × Island [Species]	3, 28	0.38	0.7648	0.90	0.4519

**Table 3:** Summary of model selection results from separate general linear models examining environmental factors in relation to different components of genital morphology. Model selection criterion for model retention was Δ AIC_c_ score <2. Model terms included piscivore density, *Gambusia* density, salinity, and turbidity (terms for species and island nested within species were included in all models)

Gonopodial feature	Model	*R*^2^	AIC_c_	Δ AIC_c_
Allometric slope (OLS)	*Gambusia* density	0.38	228.67	0.00
	Salinity	0.36	229.38	0.71
Allometric slope (RMA)	*Gambusia* density	0.34	237.16	0.00
	Salinity	0.32	238.30	1.14
Distal-tip shape	Piscivore density + Salinity	0.33	−214.17	0.00
	Piscivore density	0.25	−212.30	1.87

OLS, ordinary least squares; RMA, reduced major axis.

### Gonopodial distal-tip shape

**Figure 4:**
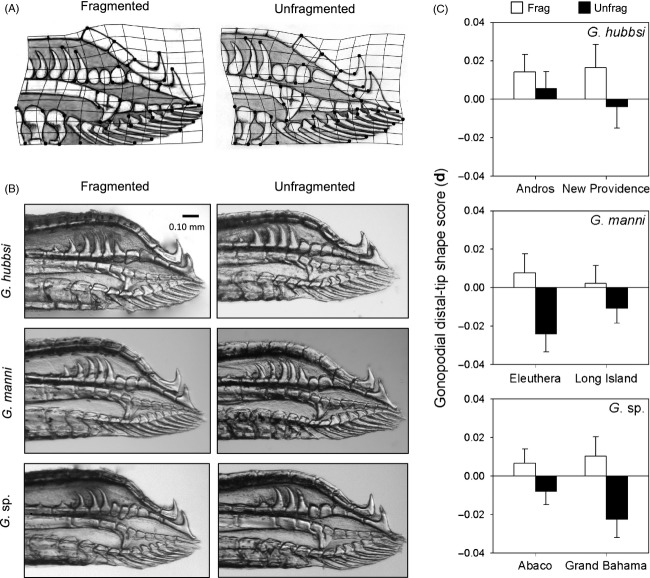
(A) Thin-plate transformation grids with illustrations overlaid onto the grids to facilitate interpretation of gonopodial distal-tip shape variation between fragmented and unfragmented tidal creeks (positive and negative scores along **d**, respectively), (B) representative photographs of gonopodial distal tips of males from each species originating from fragmented (left) and unfragmented (right) tidal creeks, and (C) least-squares means (±SE) of **d** scores from fragmented (open bars) and unfragmented (filled bars) tidal creeks across Bahamian islands.

Multivariate analysis of covariance revealed that gonopodial distal-tip shape was significantly associated with fragmentation status, multiple sources of allometry, species, and island of origin (Table 4). Based on our estimates of multivariate effect size, allometry provided the most important explanatory source, with fragmentation status representing the second most important source of variation; differences between species and islands were slightly smaller in magnitude. Combined with the lack of unique effects of fragmentation among species or islands, these results suggest a remarkably consistent pattern of differentiation in gonopodial distal-tip shape between fragmentation regimes across species and islands. Based on **d**, mosquitofish exhibited more rounded gonopodial distal tips with larger areas of soft tissue in fragmented tidal creeks, while males in unfragmented sites exhibited more elongate gonopodial distal tips with more densely positioned bony segments (Fig. 4).

**Table 4:** mancova results examining variation in gonopodial distal-tip shape across three closely related *Gambusia* species inhabiting tidal creeks across six Bahamian islands. Partial variance reflects Wilks's partial *η*^2^

Source	*F*	df	*P*	Partial variance explained (%)
Standard length	14.60	15, 175	<0.0001	55.58
Residual gonopodial surface area	2.25	15, 175	0.0064	16.18
Gonopodial distal-tip centroid size	8.34	15, 175	<0.0001	41.69
Fragmentation status (Frag)	2.56	14, 1320	0.0012	25.39
Species	2.92	28, 1849	<0.0001	17.26
Frag × Species	1.34	28, 1849	0.1123	8.92
Island [Species]	1.77	42, 2162	0.0018	13.22
Frag × Island [Species]	1.32	42, 2162	0.0836	10.53

Model selection revealed that differences in gonopodial distal-tip shape between fragmentation regimes (scores along **d**) were largely attributed to differences in piscivore density and salinity (Table 3). The top model found significant effects of piscivore density (*F*_1,34_ = 6.58, *P* = 0.0149, *η*^2^ = 13.70%) and salinity (*F*_1,34_ = 4.41, *P* = 0.0431, *η*^2^ = 9.19%). The second-best model found significant effects of piscivore density alone (*F*_1,35_ = 11.12, *P* = 0.0020, *η*^2^ = 23.57%). These environmental factors explained 65% (top model) to 67% (second model) as much variance as a term for ‘fragmentation status.’ Thus, some aspects of piscivore density and salinity appear responsible for the majority of observed differences between fragmentation regimes in gonopodial distal-tip shape.

### Gonopodial distal-tip meristics

Serrae number exhibited significant differences between species, where *G*. sp. possessed fewer serrae than either *G. hubbsi* or *G. manni* (Table 5; Table S5). Serrae number scaled positively with body size and distal-tip size and scaled negatively with relative gonopodium size (Table 5). A weak, nonsignificant trend was uncovered where *Gambusia* males tended to exhibit fewer serrae in fragmented tidal creeks (Table 5; Table S5). Number of spines on the gonopodial distal tip scaled positively with distal-tip size and exhibited a nonsignificant trend toward scaling negatively with relative gonopodium size (Table 5). No other effects were evident for the number of spines on the gonopodial distal tip (Table S5). Because neither the number of serrae nor spines differed between fragmented and unfragmented sites, we did not employ model selection to further investigate these characters.

**Table 5:** Results from separate general linear models examining variation in gonopodial distal tip meristic characters (number of serrae on ray 4p and number of spines on ray 3)

	Number of Serrae			Number of Spines		
Source	*F*	df	*P*	*F*	df	*P*
Standard length	7.44	1, 209.7	0.0069	1.91	1, 216.6	0.1689
Residual gonopodial surface area	5.88	1, 210.4	0.0162	2.97	1, 218.3	0.0861
Gonopodial distal-tip centroid size	4.78	1, 219.9	0.0298	8.32	1, 219.6	0.0043
Fragmentation status (Frag)	2.81	1, 37.98	0.1017	0.11	1, 39.62	0.7368
Species	14.75	2, 37.99	<0.0001	0.28	2, 39.64	0.7560
Frag × Species	1.06	2, 37.32	0.3568	0.38	2, 38.88	0.6850
Island [Species]	0.63	3, 37.67	0.5976	1.33	3, 39.42	0.2776
Frag × Island [Species]	1.72	3, 35.99	0.1803	0.85	3, 37.93	0.4761

## Discussion

Human activities are altering ecosystems across the globe, but how strongly, consistently, and predictably the resulting ecological shifts might cause phenotypic shifts in affected organisms remains poorly understood. Our study revealed that just 35–50 years after humans fragmented the habitats of three endemic livebearing fishes (∼70–100 generations), multiple aspects of male genital morphology exhibited consistent differences across six Bahamian islands, with some changes matching our *a priori* predictions. For instance, gonopodial distal-tip shape diverged as predicted, with more rounded tips observed in fragmented sites and more elongate and bony tips in unfragmented localities. Unexpectedly, gonopodial allometry differed consistently between fragmentation regimes, with *Gambusia* males exhibiting shallower allometric slopes in fragmented tidal creeks, while gonopodium size did not differ between fragmentation regimes as anticipated. Our findings represent the first study to report phenotypic differentiation of male genital morphology resulting from the ecological consequences of anthropogenic environmental changes.

We predicted that mosquitofish would exhibit larger relative gonopodium size in fragmented tidal creeks based on previous work in two *Gambusia* species that found males from environments experiencing reduced predation risk evolved larger gonopodia, resulting from trade-offs between natural and sexual selection under different ecological conditions (Langerhans et al. 2005). However, rather than uncovering a change in relative gonopodium size, we instead discovered a shift in allometry where the slope of gonopodial surface area versus body size repeatedly experienced a reduction subsequent to fragmentation. Based on prior work (Voje et al. 2014), we did not predict any shift in gonopodial allometry in response to the different environments caused by habitat fragmentation. So why might male *Gambusia* exhibit a shallower allometric slope in fragmented tidal creeks and more generally in sites with higher population densities and reduced salinity? Gonopodia represent a special case of genitalia because only the relatively small distal tip inserts into the female during copulation, while the entire organ is quite large relative to body size and nonretractable. Thus, popular hypotheses explaining patterns of genital allometry such as ‘one size fits all’ do not apply as neatly here compared with other systems where mechanical fit of the entire organ is required for successful fertilization (Eberhard et al. 1998; Eberhard 2009). However, gonopodium size is a target of premating sexual selection in a number of livebearing fishes (Langerhans 2011), and thus, this trait might exhibit an allometric pattern more similar to secondary sexual characters that tend to increase rapidly relative to body size (Kodric-Brown et al. 2006). This widespread trend of positive allometry for sexual ornaments and weapons traits likely exists because individuals with larger body size are typically in greater condition and can afford to invest more heavily in sexual traits than smaller individuals in poor condition (Kodric-Brown et al. 2006). Reduced *Gambusia* densities in unfragmented sites likely translate to less intense resource competition than in high-density fragmented tidal creeks. Thus, fish in unfragmented sites may experience less restrictive resource allocation trade-offs, allowing males in better condition to invest more in gonopodium size. We generally observed negative allometry of gonopodium size, with slopes closer to isometry in unfragmented sites (slope of 2 = isometry in our study). Differences in salinity might also indirectly contribute to this pattern if correlated with other unmeasured factors associated with resource availability, such as copepod or zooplankton density. Variation in population density or salinity could affect the relative strengths of selection on gonopodium and body size, potentially mediated by female preference or locomotor requirements for foraging and social/sexual behaviors, which could also contribute to allometric differences between populations (Bonduriansky and Day 2003). An additional possibility is that the observed pattern might reflect variation in the relative importance of gonopodium size during mate choice or perhaps a shift in the importance of various sexual selection mechanisms between habitats with varying conspecific densities or salinities. Understanding why ecological differences between populations may influence allometry through either phenotypic plasticity or evolutionary change merits further research attention.

Release from predation risk and reduced salinities in fragmented tidal creeks largely accounted for the observed shift in gonopodial distal-tip shape. This confirmed our predictions and mirrors recently documented evolutionary divergence in gonopodial distal-tip shape in *G. hubbsi* inhabiting environments that differ in predation risk. In both cases, populations experiencing low predation risk have more rounded distal tips with increased soft tissue compared with the more elongate and bony distal tips observed in the presence of predatory fish (Heinen-Kay and Langerhans 2013). Reduced predation risk often drastically alters the mating environment by selecting for longer copulation, more courtship, less sexual coercion and conflict, and possibly reduced postmating sexual selection (Magnhagen 1991; Magurran and Seghers 1994; Rowe et al. 1994; Heinen et al. 2013). We suggest that the more elongate and bony gonopodial tips possessed by fish in unfragmented sites facilitate more effective sperm transfer or fertilization with uncooperative females during rapid and forced copulation attempts, likely reflecting ecologically induced differences in sexual selection between fragmented and unfragmented environments. Previous work in another livebearing fish (*Poecilia reticulata*) confirms that genital shape can indeed affect sperm transfer, at least during forced copulations (Evans et al. 2011; Kwan et al. 2013). A more elongate and bony gonopodial tip may mechanically allow males to more effectively circumvent female mate choice by achieving and maintaining appropriate contact for rapid sperm transfer. Additionally, more elongate gonopodial tips could offer an advantage during sperm competition by allowing males to deposit sperm deeper inside the female's reproductive tract (van Lieshout and Elgar 2011). Under high predation risk, postcopulatory sexual selection (e.g., sperm competition, cryptic female choice) may increase in intensity to compensate for reduced premating sexual selection, increasing strength of selection on genital shape. Relaxation of sexual conflict, or possibly differences in cryptic female choice or another sexual selection mechanism, may favor the more rounded gonopodial distal tips with greater soft tissue areas observed in populations with few predators, although explanatory hypotheses are not as obvious. Why differences in salinity might affect gonopodial distal-tip shape are less clear and require further study. Salt represents an important component of many cellular processes and could affect developmental pathways; for instance, exposure to different salinity levels during development has previously been shown to underlie plastic morphological differences in stickleback body morphology (McCairns and Bernatchez 2012) and aspects of the crocodile cloaca (Kuchel and Franklin 2000). Salinity could also influence postmating physiological processes that affect fertilization, or sperm motility and life span (Elofsson et al. 2003). An additional possibility is that salinity covaries with an unmeasured (causal) environmental factor, such as density of particular resources. While it is currently unclear how resource availability might affect gonopodial distal-tip shape, previous work did find suggestive evidence for an effect of phytoplankton density on gonopodial distal-tip shape (Heinen-Kay and Langerhans 2013).

Our study revealed no differences in gonopodial meristics between fragmentation regimes other than a weak trend for fewer serrae in fragmented tidal creeks. The lack of meristic differences between *Gambusia* populations experiencing different fragmentation regimes might indicate that meristics, as opposed to multivariate shape, may not represent a target of divergent selection in this system or perhaps take longer to respond to selection. For instance, gaining or losing entire bony structures such as a spine or serra might require more substantial differentiation of developmental pathways (either via phenotypic plasticity or genetic divergence) than those for altering overall distal-tip shape. However, multiple sources of allometry were associated with both number of serrae and spines on the gonopodial distal tip, and serrae number differed among species. Although we have little functional understanding of serrae and spine number, these meristics have often proved useful in taxonomic work in *Gambusia* fishes (Greenfield 1983; Rauchenberger 1989; Langerhans et al. 2012).

Although we initially anticipated that effects of fragmentation on male genital morphology might vary among species or islands owing to a range of factors (e.g., differences in genetic variances and covariances of traits, genetic drift, variable timing of road constructions, idiosyncratic ecological effects of fragmentation across islands), our study revealed that habitat fragmentation ultimately resulted in consistent differences in multiple aspects of genital morphology. While we uncovered differences among species in some traits (distal-tip shape, serrae number) and among islands for some traits (allometry, distal-tip shape), the magnitude of differences between fragmentation regimes was typically as large or larger than these differences, and the nature of differences between fragmentation regimes did not significantly differ among species or islands. This suggests that human-induced ecological changes caused similar and strong selection on male genital morphology in fragmented tidal creeks and that population responses to similar selection were rapid, general, and repeatable.

Although we did not test for a genetic basis for differentiation in gonopodial morphology in tidal-creek mosquitofish, previous work has demonstrated a genetic basis to population differences in gonopodial distal-tip shape and gonopodium size in congeners (Langerhans et al. 2005; Heinen-Kay and Langerhans 2013). Moreover, evolutionary divergence in gonopodial morphology in *Gambusia affinis* occurred over a roughly similar timescale as examined here (Langerhans et al. 2005; Langerhans 2009). Other livebearing fish systems have witnessed rapid evolution in response to different ecological conditions with far fewer generations under different selective conditions (Reznick et al. 1990). Thus, at least some of the differences observed here may reflect rapid evolutionary changes, but future work is required to elucidate the relative importance of phenotypic plasticity and genetic divergence, either of which could reflect responses to divergent selection (West-Eberhard 2003; DeWitt and Scheiner 2004; Pfennig et al. 2010). Regardless of the source, our results indicate rapid and consistent phenotypic shifts subsequent to human-induced fragmentation—future work should examine how these phenotypic changes might alter social behaviors, demographics, or community-level ecological factors (Palkovacs et al. 2011).

Rapid differentiation of male genital morphology might result in reproductive isolation between ecologically dissimilar populations by inhibiting gene flow. Genitalia are vital for successful reproduction in internally fertilizing organisms, and mechanical and sensory incompatibilities might affect sperm transfer and fertilization success. Genital differences represent the first proposed mechanism of speciation (Dufour 1844), and to this day, taxonomists regularly use genital morphology to confirm the existence of distinct lineages (Churchill et al. 2013). Although the notion of genital differences driving reproductive isolation is controversial, it has indeed been documented in a few species such as millipedes (Wojcieszek and Simmons 2013), fruit flies (Kamimura and Mitsumoto 2012), and carabid beetles (Kubota et al. 2013). Little work has investigated the extent to which human-induced environmental change might ultimately result in speciation, and no previous work has addressed this within the context of human-induced changes in genital morphology. Whether genital differences between *Gambusia* inhabiting fragmented and unfragmented tidal creeks have led to increased levels of reproductive isolation has not yet been investigated, but would provide valuable insight into the capacity of human impacts on the environment to promote the formation of new species on ecological timescales.

How general is the phenomenon of rapid genital differentiation associated with human-induced environmental changes? Although not yet a major focus of research, the idea that variation in ecological factors can affect the mechanisms of sexual selection influencing genital evolution is gaining traction (e.g., Rowe et al. 1994; Cayetano et al. 2011; Perry and Rowe 2012; Heinen-Kay and Langerhans 2013). However, the role of human activities in ultimately driving genital differentiation has received virtually no attention prior to the present study and requires more research to understand its general significance. A handful of studies have reported male genital divergence associated with various sources of environmental variation, including host plant in *Drosophila buzzatti* (Soto et al. 2007) and a treehopper (Rodriguez and Al-Wathiqui 2011), life history selection regime in seed beetles (Cayetano et al. 2011), habitat and climate in crickets (Oneal and Knowles 2013), temperature in *Drosophila mediopunctata* (Andrade et al. 2005), and predation in livebearing fishes (Kelly et al. 2000; Jennions and Kelly 2002; Langerhans et al. 2005; Evans et al. 2011; Heinen-Kay and Langerhans 2013). Given the relationships between ecological variation, sexual selection, and genital evolution, genital diversification might represent a common outcome of human-caused environmental changes, posing significant implications for population fitness, gene flow, and possibly speciation. In the present study, we discovered that variation in predation risk, population demographics, and abiotic conditions was associated with replicated shifts in multiple aspects of male genital morphology. Altogether, our results highlight how human-induced environmental change can elicit complex and rapid phenotypic responses in affected organisms.

## Data archiving statement

Data for this study are available from the Dryad Digital Repository: http://doi.org/10.5061/dryad.4rt17.

## Supporting Information

**Focal species.** **Figure S1.** Bayesian inference phylogeny using concatenated gene sequences. **Figure S2.** Lateral photograph of a male *Gambusia hubbsi* with measurements of standard length and gonopodium surface area illustrated. **Figure S3.** Lateral photograph of a *Gambusia manni* gonopodial distal tip illustrating the 44 homologous landmarks used for geometric morphometric analysis. **Table S1.** Population sources for molecular data in the examination of genetic distinctiveness of Bahamian *Gambusia* species. **Table S2.** Sample sizes for each component of the study. For fragmentation status, F = fragmented, U = unfragmented. **Table S3.** Summary of repeatability analyses for environmental factors measured in unfragmented and fragmented tidal creeks; *r* reflects the intraclass correlation coefficient, following Lessells and Boag (1987). **Table S4.** Summary of results for separate general linear models testing for differences in environmental factors between fragmentation regimes of Bahamian tidal creeks. **Table S5.** Average number of serrae and spines on the gonopodial distal tip for the three Bahamian *Gambusia* examined in this study (adjusted for allometry; i.e., least-squares means).
