# Supplementary material for: Human-caused habitat fragmentation can drive rapid divergence of male genitalia
Source: Evol Appl. 2014 Oct 31;7(10):1252–67. doi: 10.1111/eva.12223 (PMC4275096; doi:10.1111/eva.12223)

Supporting Information for:
Human-caused habitat fragmentation can drive rapid divergence of male genitalia

Justa L. Heinen-Kay, Holly G. Noel, Craig A. Layman, and R. Brian Langerhans

Focal species

Ongoing phylogenetic and taxonomic work across the genus *Gambusia* has clearly revealed three endemic species of *Gambusia* in The Bahama Archipelago (R. B. Langerhans, O. Dominguez, C. Pedraza-Lara, M. E. Gifford, I. Doadrio, unpublished data). While two *Gambusia* species have been described from The Bahamas, a third species not yet described also occurs there. Fink (1971) and Rauchenberger (1989) suggested that the range of *Gambusia manni* is limited to two brackish lakes on New Providence Island (Lake Cunningham and Lake Killarney), while *G. hubbsi* inhabits many Bahamian islands. Extensive collections by R.B. Langerhans across The Bahamas over the past 12 years, and inspection of material from museums, has instead found evidence suggesting that (1) *G. manni* represents a widespread species, apparently inhabiting all islands other than Bimini, Berry Islands, and Andros within the Great Bahama Bank, as well as isolated carbonate platforms in the southeastern Bahamas (although it indeed only occurs within New Providence in those two brackish lakes), (2) *G. hubbsi* occurs on Bimini, Berry Islands, Andros, and New Providence within the Great Bahama Bank, and (3) a yet unnamed species, *G.* sp., occurs on the islands of the Little Bahama Bank (Grand Bahama, Abacos). An upcoming phylogenetic analysis of Bahamian *Gambusia*, within the context of the entire genus, will be published elsewhere (R. B. Langerhans, O. Dominguez, C. Pedraza-Lara, M. E. Gifford, I. Doadrio, unpublished data); and a formal description of *G.* sp. from the Little Bahama Bank awaits future study. Here we present molecular genetic evidence for the existence of three divergent, reciprocally monophyletic *Gambusia* species in the Bahamas.

To assess genetic divergence and phylogenetic relationships among the three Bahamian *Gambusia* species, we obtained mtDNA and nDNA gene sequences for Bahamian *Gambusia* and four outgroup taxa (Table S1). We examined samples of Bahamian *Gambusia* from a total of 17 coastal localities across eight islands. For mtDNA, we sequenced fragments of the NADH subunit 2 gene (ND2) and the cytochrome *b* gene (cyt *b*). For nDNA, we sequenced fragments of the first intron of the S7 ribosomal protein gene (S7) and the recombination activation gene 2 (RAG2). PCR primers and conditions for ND2, cyt *b*, and S7 followed Langerhans et al. (2012). For RAG2, we used the primers RAG2-F (5′ - GAC CCC GAG YGY TAC CTC ATC C - 3′) and RAG2-R (5′ - TCG GTG GAG TAG TAA GGC TCC CA - 3′). PCR conditions included an initial denaturation at 95°C for 180 s followed by 30 cycles of denaturation at 95°C for 30 s, annealing at 57°C for 30 s, and extension at 72°C for 90 s; concluding with a final extension at 72°C for 240 s. Aligned fragment lengths were: ND2: 1031 bp, cyt *b*: 402 bp, S7: 745 bp (with 4 indels), and RAG2: 972 bp. Sequences were aligned by eye. We generated 58 new sequences for this analysis, and used six previously published sequences from GenBank (U18209, U18211, U18214, U18228, KF704052, KF704055). All new sequence data have been deposited in GenBank (KM658333-KM658396).

We inferred phylogenetic relationships from concatenated DNA sequences (3150 bp) using maximum-likelihood (ML) and Bayesian inference (BI) approaches. A total of eight data partitions were employed: one for each codon position of the ND2 and cyt *b* genes, one for S7, and one for RAG2. Maximum-likelihood phylogenetic relationships were estimated using PAUP 4.0b10 (Swofford 2003), with the optimal maximum-likelihood model of DNA sequence

evolution determined using the Akaike information criterion (AIC) with jModelTest2 (Guindon and Gascuel 2003; Darriba et al. 2012). Rates were optimized separately for each data partition. The ML heuristic search employed 10 replicate random-sequence stepwise additions for starting trees and tree bisection and reconnection branch swapping. To estimate support for nodes in the ML trees, 100 bootstraps of sequence data were generated, preserving partitioning structure using RAxML 7.03 (Stamatakis 2006). We estimated BI relationships using MrBayes 3.2.1 (Ronquist et al. 2012), with the optimal maximum-likelihood model of sequence evolution determined as above for each of the eight data partitions. We performed partitioned mixed-model Bayesian analyses, where each data partition was assigned its own evolutionary model, with model parameter values being unlinked among partitions assigned the same molecular evolutionary model. MrBayes 3.2.1 was run for 5,000,000 generations, sampling trees every 100 generations, with the lower 25% of trees discarded as burn-in (after checking that stationarity was reached) for computation of a 50% majority-rule consensus tree. We calculated support values for inferred clades using Bayesian posterior probabilities.

Phylogenetic analysis revealed strong support for three, reciprocally monophyletic species of *Gambusia* in the Bahamas (Fig. S1). While all three clades are well supported, interrelationships among the three species are not totally clear; although, current results suggest a sister relationship between *G. hubbsi* and *G. manni*. To provide rough estimates of divergence times between Bahamian *Gambusia* species, the 95% CI of divergence time estimates of Hrbek et al. (2007) for a node within the genus *Gambusia* were used to construct a molecular clock for the regions of ND2 and cyt *b* examined here (the only overlapping gene regions between the two studies) (see Langerhans et al. 2012). Based on these molecular clock estimates, *G.* sp. diverged from the clade of *G. hubbsi* and *G. manni* approximately 1.68-4.83 million years ago, while *G. hubbsi* and *G. manni* diverged from one another approximately 1.30-2.36 million years ago. All six islands examined in this study (see main text) were represented in this phylogenetic analysis. We use the species designations indicated here throughout the study.

References

Darriba, D., G. L. Taboada, R. Doallo, and D. Posada. 2012. jModelTest 2: more models, new heuristics and parallel computing. Nature Methods **9**: 772.

Fink, W. L. 1971. A revision of the *Gambusia puncticulata* complex (Pisces: Poeciliidae). Publications of the Gulf Coast Research Laboratory Museum **2**: 11-46.

Guindon, S., and O. Gascuel. 2003. A simple, fast and accurate method to estimate large phylogenies by maximum-likelihood. Systematic Biology **52**: 696-704.

Langerhans, R. B., M. E. Gifford, O. Domínguez-Domínguez, D. García-Bedoya, and T. J. DeWitt. 2012. *Gambusia quadruncus* (Cyprinodontiformes: Poeciliidae): a new species of mosquitofish from East-central Mexico. Journal of Fish Biology **81**: 1514-1539.

Lessells, C. M., and P.T. Boag. 1987. Unrepeatable repeatabilities—A common mistake. Auk **104**: 116-121.

Rauchenberger, M. 1989. Systematics and biogeography of the genus *Gambusia* (Cyprinodontiformes: Poeciliidae). American Museum Novitates **2951**: 1-74.

Ronquist, F., M. Teslenko, P. van der Mark, D. L. Ayres, A. Darling, S. Hohna, et al. 2012. MrBayes 3.2: Efficient Bayesian phylogenetic inference and model choice across a large model space. Systematic Biology **61**: 539-542.

Stamatakis, A. 2006. RAxML-VI-HPC: maximum likelihood-based phylogenetic analyses with thousands of taxa and mixed models. Bioinformatics **22**: 2688-2690.

Swofford, D. L. 2003. PAUP*: phylogenetic analysis using parsimony (*and other methods). Sinauer Associates, Sunderland, MA.

Table S1 Population sources for molecular data in the examination of genetic distinctiveness of Bahamian *Gambusia* species.

Species	Location	Collector(s)	Genes Sequenced
G. hubbsi	Davis Creek, Andros Island, Great Bahama Bank	R.B. Langerhans	ND2
G. hubbsi	Mastic Point Creek, Andros Island, Great Bahama Bank	R.B. Langerhans	ND2
G. hubbsi	Independence Park Creek, Andros Island, Great Bahama Bank	R.B. Langerhans	ND2
G. hubbsi	Thompson/Scott Creek, Andros Island, Great Bahama Bank	R.B. Langerhans	ND2, cyt *b*, S7, RAG2
G. hubbsi	Mangrove Lake, Andros Island, Great Bahama Bank	R.B. Langerhans, L. Beckman	ND2, cyt *b*, S7
G. hubbsi	Millar Creek, New Providence Island, Great Bahama Bank	R.B. Langerhans, C.A. Layman	ND2
G. hubbsi	Lake Killarney, New Providence Island, Great Bahama Bank	R.B. Langerhans, C.A. Layman	ND2
G. hubbsi	Adelaide Creek, New Providence Island, Great Bahama Bank	R.B. Langerhans, C.A. Layman	ND2, cyt *b*
G. hubbsi	Defense Creek, New Providence Island, Great Bahama Bank	R.B. Langerhans, E.M.A. Hassell, K. Quigley	ND2, cyt *b*, S7, RAG2
G. manni	Lake Cunningham, New Providence Island, Great Bahama Bank	R.B. Langerhans, C.A. Layman	ND2, cyt *b*, S7, RAG2
G. manni	Lake Cunningham, New Providence Island, Great Bahama Bank	R.B. Langerhans, C.A. Layman	ND2, S7, RAG2
G. manni	Burrows Pond, Eleuthera Island, Great Bahama Bank	C. McKinney Lambert	ND2, cyt *b*, S7, RAG2
G. manni	Gordon's Creek, Long Island, Great Bahama Bank	R.B. Langerhans, E.M.A. Hassell, K. Quigley	ND2, cyt *b*, S7, RAG2
G. manni	Norman's Cay, Exuma Cays, Great Bahama Bank	R.B. Langerhans, C.A. Layman	ND2, cyt *b*
G. manni	Hot Creek, Great Exuma Island, Great Bahama Bank	R.B. Langerhans, C.A. Layman	ND2, cyt *b*, S7, RAG2
G. manni	Gold Dust Pond, San Salvador Island, Isolated Bank	M. Barton	cyt *b*
G. sp.	Twisted Bridge Creek, Abaco Island, Little Bahama Bank	C.A. Layman	ND2, cyt *b*, S7, RAG2
G. sp.	Expansive Creek, Grand Bahama Island,	R.B. Langerhans, C.A. Layman	ND2, cyt *b*, S7, RAG2

	Little Bahama Bank		
G. hispaniolae	Duverge stream, Dominican Republic	R.B. Langerhans	ND2, cyt *b*, S7, RAG2
G. nicaraguensis	Laguna de Karata, Nicaragua	W.A. Matamoros	ND2, cyt *b*, S7, RAG2
G. rhizophorae	Matheson Hammock, Florida	R.B. Langerhans, C. Ruehl	ND2, cyt *b*, S7, RAG2
G. wrayi	Bluefields stream, Jamaica	R.B. Langerhans	ND2, cyt *b*, S7, RAG2

Table S2 Sample sizes for each component of the study. For fragmentation status, F=fragmented, U=unfragmented.

Species	Island	Frag status	Population	Gonopodium size (n)	Gonopodial distal tip (n)
G. hubbsi	Andros	F	Fresh Creek Back Up	10	3
		U	Independence Park	10	4
			Red Bays Pond	5	3
			Thompson / Scott	10	6
			Cargill Creek	10	3
			Davy Creek	10	6
			Fresh Creek Twin Lakes	10	3
			Stafford Creek North	10	5
	New Providence	F	Adelaide Up	10	6
		U	Fox Hill Creek Up	7	4
			Defense Creek	9	5
			Fox Hill Creek Down	7	3
			South Beach Creek	3	2
G. manni	Eleuthera	F	John Miller	10	5
		U	Princess Cay	10	5
			Tarpum Bay	9	5
			Airport	10	10
			Cape Eleuthera	10	4
			Cruise Ship	10	6
	Long Island	F	Airport Creek	9	2
		U	Gordon's Beach	10	5
			Stella Maris	8	4
			Two Sisters	10	4
			Clarence Creek	10	5
			Cliff Creek	16	16
			Glinton's Creek	10	5
			Gordon's Creek	10	7
G. sp.	Abaco	F	Camp Abaco	15	15
		U	Crossing Rocks	9	4
			Double Blocked Up	10	3
			Indian River East	9	4
			Stinky Pond	10	5
			Blue Holes Creek	11	7
			Cherokee Creek	16	15
			Sand Bar	4	3
			Treasure Cay	10	8
			Twisted Bridge	10	5
	Grand Bahama	F	Crumbling Road	10	5
		U	Jellyshell West	10	6
			Rainy Blocked Creek	8	3
			Blue Holes Creek	7	5
			Empty House Creek	9	6
			Expansive Creek	9	5

Table S3 Summary of repeatability analyses for environmental factors measured in unfragmented and fragmented tidal creeks; *r* reflects the intraclass correlation coefficient, following Lessells and Boag (1987).

Environmental factor	# times measured per site	Timescale	# unfrag sites	# frag sites	Total # measurements	*r*	*P*
Piscivore density	3-7	2 years	5	8	72	0.83	<0.0001
Gambusia density	2	9 months	5	7	24	0.72	0.0023
Salinity	2-19	12 years	11	13	225	0.92	<0.0001
pH	2-5	10 years	8	11	55	0.56	<0.0001
Turbidity	2-3	4 years	7	11	38	0.46	0.0146
Dissolved oxygen	2-6	10 years	8	12	56	0.32	0.0156

Table S4 Summary of results for separate general linear models testing for differences in environmental factors between fragmentation regimes of Bahamian tidal creeks. Models included five terms (fragmentation status, species, island nested within species, fragmentation status × species, and fragmentation status × island nested within species). We present here only results for the effect of fragmentation regime, as we were specifically interested in whether these ecological variables exhibited consistent differences between fragmented and unfragmented tidal creeks.

Source	*F*	df	*P*
Piscivore density	46.10	1,31	<0.0001
Gambusia density	25.00	1,30	<0.0001
Salinity	7.50	1,31	0.0101
Turbidity	3.59	1,31	0.0674
pH	1.56	1, 31	0.2209
Dissolved oxygen	0.19	1, 31	0.6688

Table S5. Average number of serrae and spines on the gonopodial distal tip for the three Bahamian *Gambusia* examined in this study (adjusted for allometry; i.e., least-squares means). For fragmentation status, F=fragmented, U=unfragmented.

Species	Island	Frag Status	Ray 4p serrae number	Ray 3 spine number
G. hubbsi	Andros	F	5.18	9.77
		U	5.03	9.75
	New Providence	F	5.05	9.93
		U	5.35	9.63
G. manni	Eleuthera	F	4.81	9.86
		U	5.41	9.54
	Long Island	F	5.24	9.99
		U	5.44	10.17
G. sp.	Abaco	F	4.43	9.58
		U	4.79	9.87
	Grand Bahama	F	4.70	9.96
		U	4.49	9.88

Fig. S1 Bayesian inference phylogeny using concatenated gene sequences. Numbers above and below branches indicate Bayesian inference posterior probabilities and maximum likelihood bootstrap percentages for each node, respectively. The geographical locality of each sample is given in parentheses. The well-supported nodes for the three Bahamian *Gambusia* species are denoted with red circles.

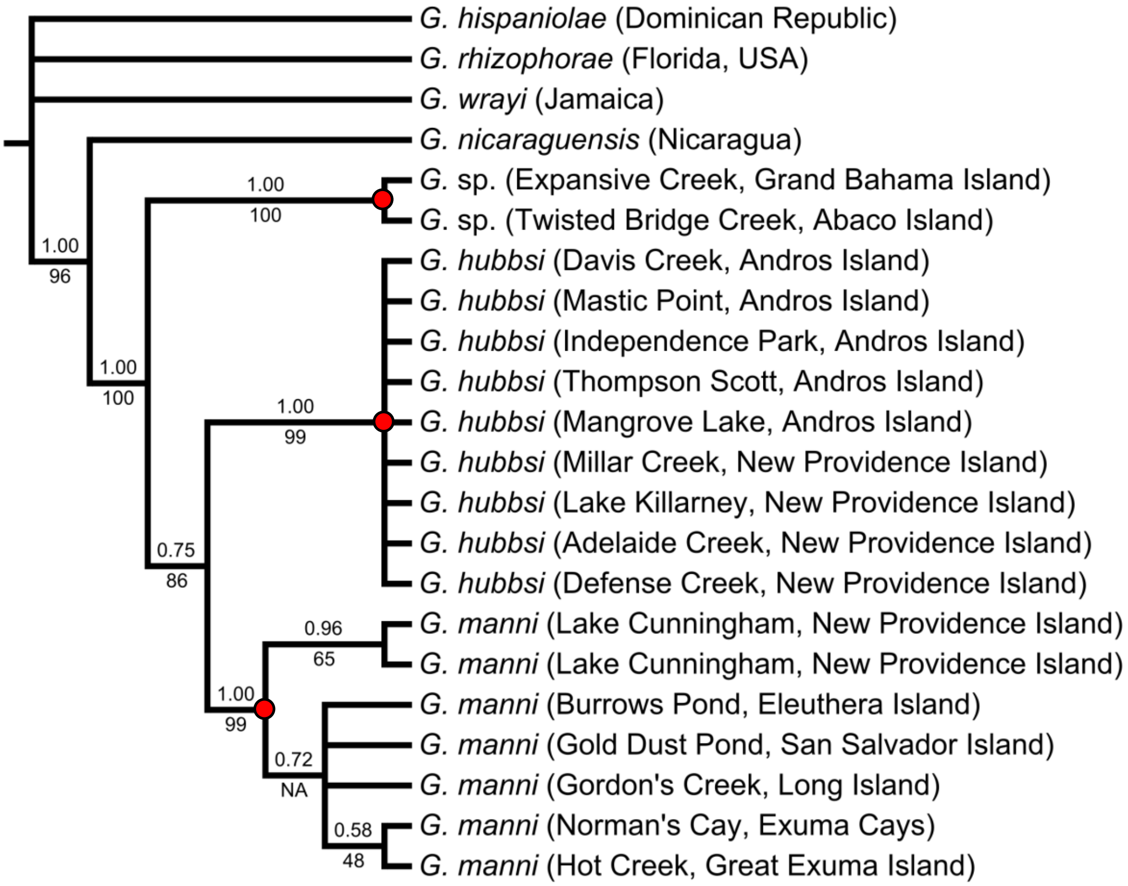

Fig. S2 Lateral photograph of a male *Gambusia hubbsi* with measurements of standard length and gonopodium surface area illustrated.

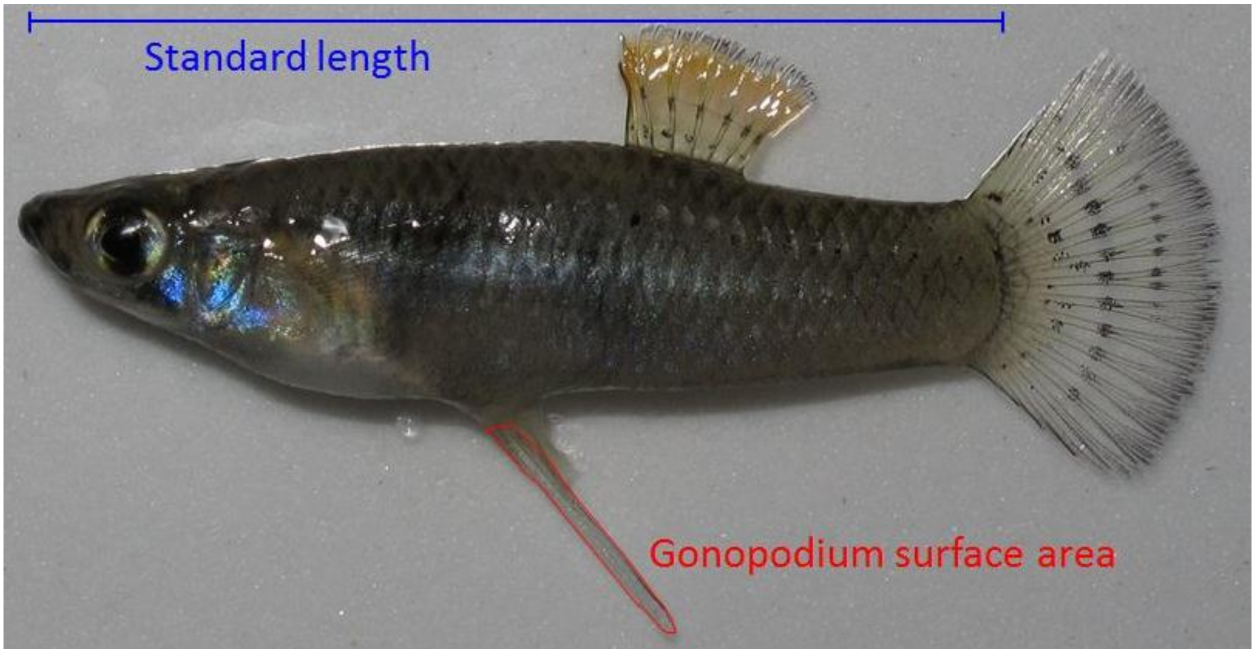

Fig. S3 Lateral photograph of a *G. manni* gonopodial distal tip illustrating the 44 homologous landmarks used for geometric morphometric analysis. Textual descriptions of landmarks provided in Table S3 of Heinen-Kay and Langerhans (2013; note that landmarks 38-44 in this study correspond to landmarks 41-47 in the previous study).

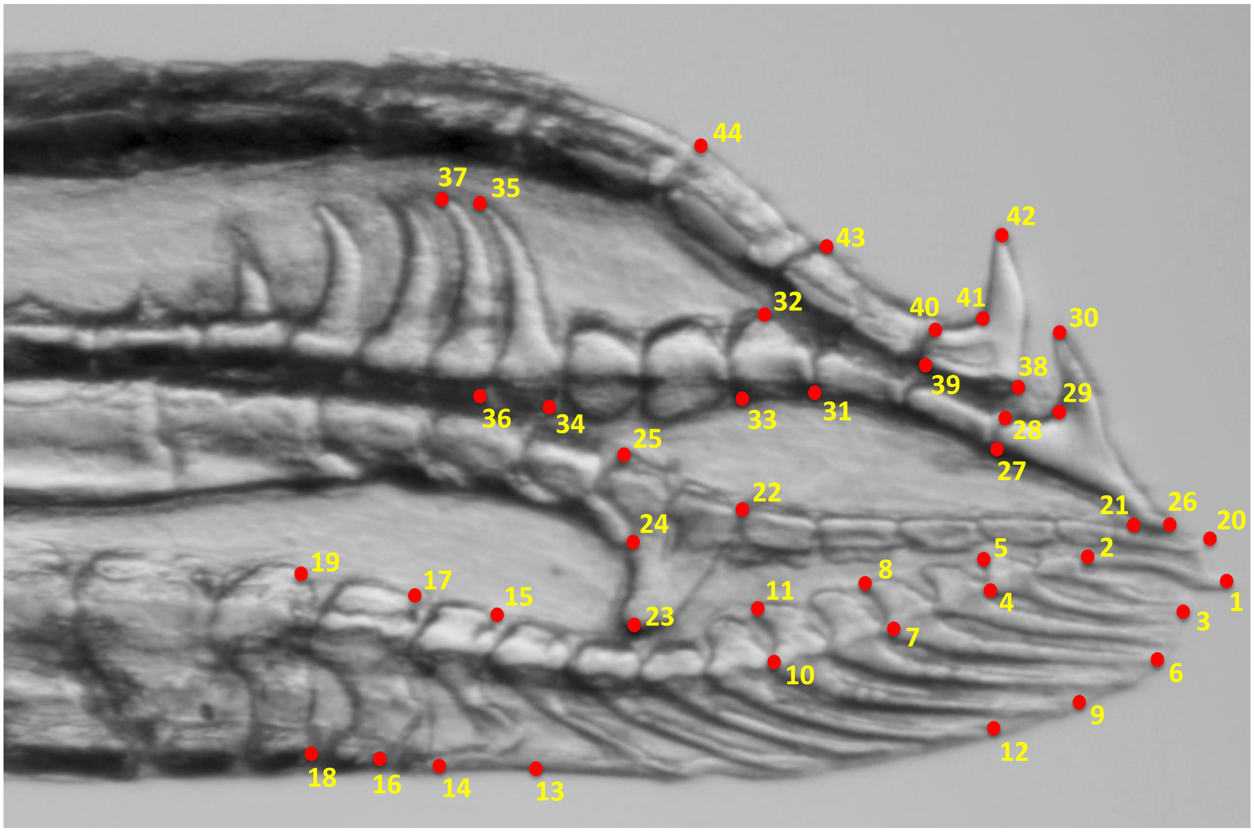

Supplement: Supplementary file 1 [file eva0007-1252-sd1.pdf]
